# Unified Accurate Attitude Control for Dual-Tiltrotor UAV with Cyclic Pitch Using Actuator Dynamics Compensated LADRC

**DOI:** 10.3390/s22041559

**Published:** 2022-02-17

**Authors:** Zexin Wang, Yingxun Wang, Zhihao Cai, Jiang Zhao, Ningjun Liu, Yanqi Zhao

**Affiliations:** 1 School of Automation Science and Electrical Engineering, Beihang University, Beijing 100191, China; wangzx@buaa.edu.cn (Z.W.); zhaoyanqi@buaa.edu.cn (Y.Z.); 2 Institute of Unmanned System, Beihang University, Beijing 100191, China; wangyx@buaa.edu.cn (Y.W.); liuningjun@buaa.edu.cn (N.L.)

**Keywords:** dual-tiltrotor, linear active disturbance rejection control (LADRC), UAV, unified controller

## Abstract

This paper proposes a unified attitude controller based on the modified linear active disturbance rejection control (LADRC) for a dual-tiltrotor unmanned aerial vehicle (UAV) with cyclic pitch to achieve accurate attitude control despite its nonlinear and time-varying characteristics during flight mode transitions. The proposed control algorithm has higher robustness against model mismatch compared with the model-based control algorithms. The modified LADRC utilizes the state feedbacks from the onboard sensors like IMU and Pitot tube instead of the mathematical model of the plane. It has less dependency on the accurate dynamics model of the dual-tiltrotor UAV, which can hardly be built. In contrast to the original LADRC, an actuator model is integrated into the modified LADRC to compensate for the non-negligible slow rotor flapping dynamics and servo dynamics. This modification eliminates the oscillation of the original LADRC when applied on the plant with slow-response actuators, such as propeller and rotors of the helicopter. In this way, the stability and performance of the controller are improved. The controller replaces the gain-scheduling or the control logic switching by a unified controller structure, which simplifies the design approach of the controller for different flight modes. The effectiveness of the modified LADRC and the performance of the unified attitude controller are demonstrated in both simulation and flight tests using a dual-tiltrotor UAV. The attitude control error is less than ±4° during the conversion flight. The control rising time in different flight modes is all about 0.5 s, despite the variations in the airspeed and tilt angle. The flight results show that the controller guarantees high control accuracy and uniform control quality in different flight modes.

## 1. Introduction

A tiltrotor unmanned aerial vehicle (UAV) utilizes tiltable rotors to change the direction of the thrust for high-speed cruise and vertical takeoff and landing. It is an important category of vertical takeoff and landing (VTOL) UAVs that has gained fast-growing popularity in both academia and industry [1,2,3,4,5,6]. According to the number of rotors, tiltrotor UAVs can be divided into two categories, dual-tiltrotor UAVs and multi-tiltrotor UAVs [7]. Apart from the different number of rotors, the main difference between the dual-tiltrotor UAVs and multi-tiltrotor UAVs is that the former uses two helicopter rotors with collective and cyclic pitch to provide control forces and moments, such as V-247 and Eagle eye [6]. Multi-tiltrotor UAVs, on the other hand, have multiple rotors mounted in front of and behind the center of gravity to provide control forces and moments, just like traditional multi-rotor UAVs. In the cruise period, their rotors that do not provide thrust become dead weight and reduce the efficiency of cruise flight [7]. Owing to the high efficiency in hover and cruise flight, the dual-tiltrotor UAV is a promising configuration for the high performance VTOL UAV [4,6]. 

However, the accurate dynamics model of the dual-tilt rotor UAV can hardly be built. This means that the classical model-dependent control algorithms can hardly achieve ideal performance. The rotor downwash of the dual-tiltrotor UAV causes nonlinear and time-varying aerodynamic interference between the rotor and wings in the hover and transition flight, which can hardly be modeled theoretically [8,9]. Moreover, the high order rotor flapping dynamics and rotor-body coupling dynamics make it more difficult to build an accurate dynamics model for the controller design [10,11]. Thus, it is a challenge to design a safe and robust flight control system for the dual-tiltrotor UAV in the presence of high nonlinearity, time-varying disturbances, and model uncertainties. Further, it is possibly one of the reasons for the small number of dual-tiltrotors developed so far.

In recent years, many attempts have been made to apply both classical and advanced control algorithms to solve the flight control problems of VTOL UAVs [7]. The classical PID controller [12] and the linear quadratic regulator (LQR) [13] are the most common linear control laws applied in VTOL UAVs. Although linear control laws are easy to implement and are computationally efficient, their performance degrades when operating far from the equilibrium point, which is encountered during the transition flight. Therefore, gain-scheduling [14], nonlinear dynamic inversion (NDI) [15], model predictive control (MPC) [16], and separated controllers that have different controllers for different flight modes are used to improve the performance during the transition. However, they still require the priori model of the UAV and are sensitive to model uncertainties. Robust μ synthesis or linear quadratic Gaussian controllers [17], as well as model reference adaptive control (MRAC), are applied to reduce the model dependence [18]. However, the performance of the robust controller is conservative and it is hard to tune the high-order controller when applied on the real tilt-rotor UAV. Although these control algorithms partially solve the flight control problem of the tiltrotor UAV, they rely heavily on accurate plant models.

Other options to reduce the sensitivity of model uncertainties are the incremental nonlinear dynamic inversion (INDI) [19] and active disturbance rejection control (ADRC) [20]. INDI replaces the model information by the measurement or estimation of the linear or angular accelerations of the UAV. The measurement or estimation of the acceleration has inevitable noise and bias [21]. ADRC uses an extended state observer (ESO) to estimate the model uncertainty and external disturbances that are then compensated by the controller [22]. It has strong robustness against model uncertainties and external disturbances. The original nonlinear ADRC is simplified to the linear active disturbance rejection control (LADRC) [23], which enables the theoretical tuning of its parameters according to the required controller bandwidth. Thus, LADRC is a promising approach to solve the nonlinear time-varying control problem of the dual-tiltrotor UAV.

However, the original LADRC controller design process assumes that the actuator dynamics are fast enough and are ignored [22,23]. In practice, dual-tiltrotor UAVs are driven by helicopter rotors and aerodynamic control surfaces that have limited bandwidth. Thus, the rotor’s relatively slow flapping dynamics and servo dynamics have non-negligible effects on the stability of the LADRC closed-loop control systems. The drawback of ignoring the actuator dynamics can be seen from some existing applications of the original ADRC on UAVs [24,25,26]. These ADRC controllers are designed without considering the actuator dynamics. Consequently, to maintain the stability of the closed-loop system in UAV applications, the bandwidth of ESO is lowered to unreasonable values [23]; otherwise, the system will oscillate or even diverge. The relative analysis can be found in [27]. Thus, the original LADRC needs a modification to compensate for the non-negligible rotor flapping dynamics to improve the stability and performance in the application on the dual-tiltrotor UAV. 

In this work, an LADRC-based unified accurate attitude controller for the dual-tiltrotor UAV is proposed to counteract its model uncertainty, nonlinearity, and time-varying characteristics. It has the ability to achieve unified control performance in different flight modes. The controller is based on the modified LADRC algorithm that compensates for the dynamics of the actuators, such as the rotor flapping dynamics and servo dynamics of the dual-tiltrotor UAV. The rotor flapping dynamics model is obtained by the system identification and then integrated into the modified LADRC, so that the ESO bandwidth can be set to a reasonable value and improved controller performance can be achieved [27]. The unified controller enables continuous control over the entire flight envelope, and the gain-scheduling or the switching between different controllers are unnecessary. Simulation and flight tests are conducted to demonstrate the effectiveness of the controller. 

The outline of this paper is as follows. Section 2 introduces the dynamics model of the studied dual-tiltrotor UAV. In Section 3, the unified attitude controller and the modified LADRC are illustrated. Section 4 and Section 5 present the simulation and flight test results, respectively. Conclusions are drawn in Section 6.

## 2. Modeling

### 2.1. Configuration Descriptions

**Figure 1:**
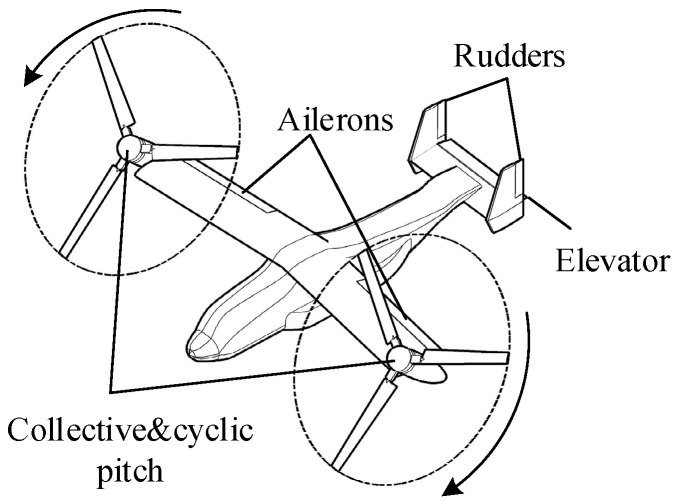
Aircraft configuration.

The configuration of the studied dual-tiltrotor UAV is shown in Figure 1. The studied dual-tiltrotor UAV is powered by two motor-driven helicopter rotors mounted at the tip of the wing. Its rotors have the same collective pitch and longitudinal cyclic pitch mechanisms as the helicopter. The two rotor nacelles can tilt forward from the helicopter mode to the fixed-wing mode symmetrically. Apart from the rotors and nacelles, the main body of the UAV has a normal configuration of the fixed-wing aircraft, which has ailerons, elevators, and rudders, as shown in Figure 1. The controls of the aircraft are listed in Table 1. The rotors and aerodynamic surfaces can both generate control moments in the roll, pitch, and yaw channels. Thus, the studied dual-tiltrotor UAV has redundant controls for the longitudinal and latitudinal control channels.

### 2.2. Dynamics Model

In the absence of experimental data, accurate dynamics models for dual tilt rotor UAVs are difficult to establish by theoretical methods alone. For example, the rotor downwash of the dual-tiltrotor UAV causes nonlinear and time-varying aerodynamic interference between the rotor and wings, which can hardly be modeled theoretically [8,9]. Moreover, the high order rotor flapping dynamics and rotor-body coupling dynamics make it more difficult to build an accurate dynamics model for the dual-tilt rotor UAV [10,11]. On the other hand, an accurate dynamics model is not necessary owing to the high robustness of LADRC [20], which is one of the important reasons for designing the controller based on LADRC. The unmodeled dynamics are considered as model uncertainty and are compensated by the LADRC controller.

Therefore, a simplified dynamics model is built for controller design and preliminary verification using the well-developed methods [28]. The rotor dynamics that contain sensitive controller parameters are calibrated by the system identification.

**Figure 2:**
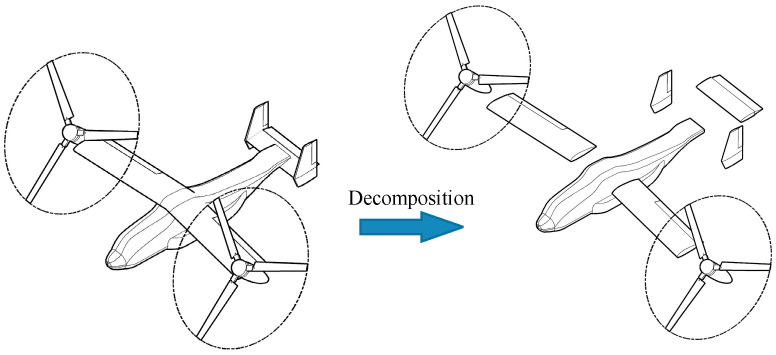
Aerodynamic components of the aircraft.

A component-based method is used to theoretically model the aerodynamics of the tiltrotor UAV [29]. In the model, the aircraft is divided into several components that generate aerodynamic forces and moments, as shown in Figure 2. They are modeled separately and then assembled along with the gravity to estimate the forces and moments experienced by the aircraft.

**Figure 3:**
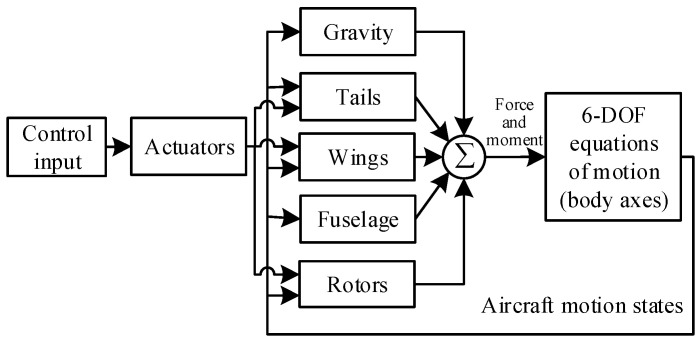
Model structure.

The structure of the UAV model is shown in Figure 3. The interaction of the rotors and wings is neglected and treated as the model uncertainty, as it can hardly be modeled precisely by theoretical approaches. Further, the design of the LADRC controller does not require such a detailed model, which is one of the advantages of the LADRC control algorithm. 

Well-established modeling methods are used to calculate the forces and moments produced by the fixed-wing part, including the wings, tails, and the fuselage [28,29]. As for the rotors that have flapping dynamics, a quasi-static closed-form method is utilized, which has been applied in the industry with good results [29]. In order to simplify the rotor force and moment equations and obtain reduced-order linear flapping dynamics for the controller design, the following assumptions are used [30]:

(1) Owing to the relatively small size of the rotor, its blade is rigid in bending and torsion and its flapping angle is small (<10 deg).
(2) The rotor-induced velocity is uniformly distributed.
(3) The lead-lag motion, which produces significantly smaller forces on the rotor hub than the flapping, is ignored.
(4) The quasi-static solution to the flapping equation is a periodic function that can be expressed as the Fourier series with the second and higher harmonics ignored.

With the above assumptions, the rotor’s flapping equation of motion reduces to the first-order rotor tip-path-plane dynamics [30]:

(1) $$\begin{array}{l} \tau_{f}{\dot{a}}_{L(R)}=-a_{L(R)}-\tau_{f}q-A_{b}b_{L(R)}+B_{1L(R)}\\ \tau_{f}{\dot{b}}_{L(R)}=-b_{L(R)}-\tau_{f}p-B_{a}a_{L(R)} \end{array}$$

where $a_{L(R)}$ and $b_{L(R)}$ represent the longitudinal and lateral flapping angles of the left or right rotors, respectively; $\tau_{f}$ is the rotor-flap time constant; $A_{b}$ and $B_{a}$ are the rotor cross-coupling coefficients; p and q are the roll and pitch angular speed in the body axes; and $B_{1L(R)}$ is the longitudinal cyclic pitch angle of the left or right rotor. It can be seen from (1) that the rotor flapping dynamics are coupled with the aircraft’s body angular dynamics.

The rotor forces and moments are calculated in their reference axes based on the tip-path-plane dynamics using the combined blade-element/momentum theory analysis [31]. The tilting of the rotor’s tip-path-plane generates control moments and forward/backward control forces that are the components of the rotor’s thrust. Then, they are transformed back to the body axes. All of the forces and moments of each component are summed up and fed to the six-degrees-of-freedom equations of motion [28] to calculate the aircraft’s motion states.

### 2.3. Identification of Rotor-Body Coupling Dynamics

The rotor-body coupling dynamics model contains the time constant of the rotor dynamics and the control effectiveness of the rotor, which are crucial parameters for the modified LADRC controller to eliminate the oscillation caused by the unsynchronized feedback signals [27]. The original LADRC controller design process assumes that the actuator dynamics are fast enough and are ignored. However, the response of helicopter rotors to the collective and cyclic pitch control is relatively slow owing to the inertia and aerodynamics of the blades, which must be considered in the LADRC controller design. Thus, the accurate rotor-body coupling dynamics are indispensable in both the modeling and the LADRC controller design. 

**Figure 4:**
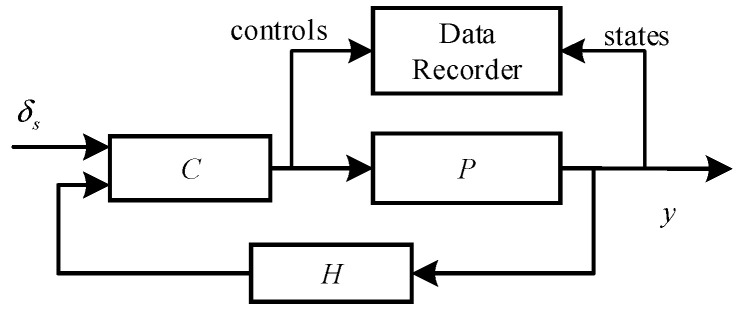
Closed-loop system for system identification.

In order to obtain relatively accurate rotor-body coupling dynamics, in addition to theoretical analysis, system identification is performed. The closed-loop system for system identification is shown in Figure 4, where C is the controller, P represents the aircraft, H is the sensor, y is the states of motion, and $\delta_{s}$ is the attitude command.

**Figure 5:**
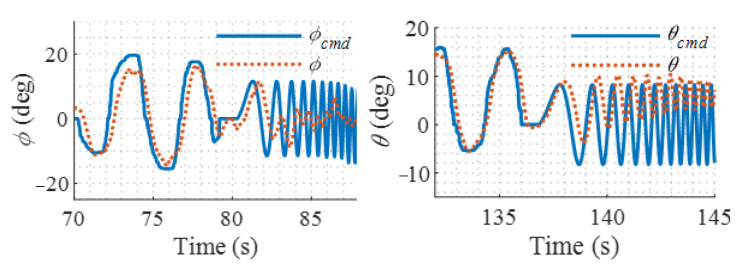
Frequency sweep responses.

Automated frequency-sweep attitude command is input into the attitude controller, which is an excitation signal with sufficient spectral richness for the studied dual-tiltrotor UAV. It has a bandwidth of 1 rad/s to 30 rad/s and a duration of 12 s. The key rotor parameters are then identified from the recorded input and output signals using a suitable identification model for the lateral and longitudinal rotor-body coupling dynamics. In the helicopter mode, flight tests for system identification in the roll and pitch channels are conducted and the UAV is controlled by a primary PID hover controller. As for the yaw channel, the aircraft’s directional response to the rotor is severely attenuated by the vertical stabilizer and the fuselage, so its data are of low quality and ignored. The frequency sweep commands and attitude responses are shown in Figure 5.

**Figure 6:**
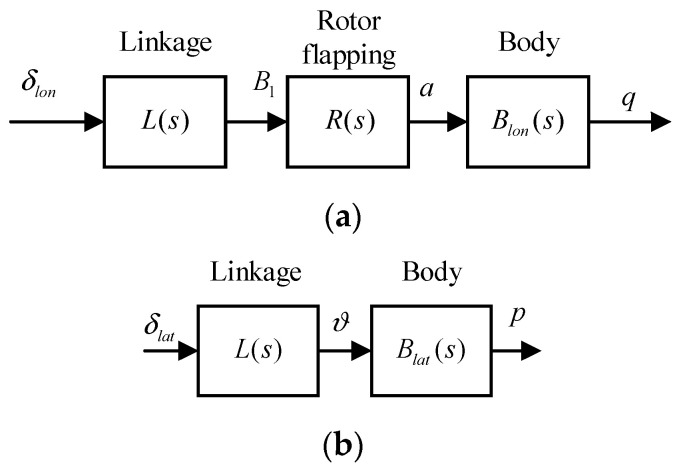
Identification model. (**a**) Longitudinal model; (**b**) Lateral model.

The rotor-body coupling model for the system identification is simplified to a cascade single-input-single-output form. It contains the least number of model parameters that can well approximate the response of the aircraft [29,31]. The longitudinal and lateral models are shown in Figure 6, where $\delta_{lon}$ is the symmetric longitudinal cyclic pitch command, $\delta_{lat}$ is the asymmetric collective pitch command, $B_{1}$ is the longitudinal cyclic pitch angle, ϑ is the collective pitch angle, a is the longitudinal flapping angle, and p and q are the aircraft’s roll and pitch angular speeds. As for the lateral model shown in Figure 6b, the rotor flapping transfer function is removed, as the roll control only engages the asymmetric deflections of the collective pitch and it does not cause the tilting of the tip-path-plane. 

These models consist of a linkage model L(s), a first-order rotor flapping dynamics model R(s), a quasi-steady body longitudinal model $B_{lon}(s)$, and a lateral model $B_{lat}(s)$. The rotor flapping dynamics model is the simplified form of (1) that neglects the cross-coupling terms. The simulation and flight test results show that the simplified rotor flapping dynamics model is adequate for the LADRC to achieve satisfactory performance. Their transfer functions are as follows:

(2) $$\begin{array}{l} L(s)=\frac{B_{1}(s)}{\delta_{lon}(s)}=e^{-\tau_{l}s},R(s)=\frac{a(s)}{B_{1}(s)}=\frac{1}{\tau_{f}s+1},\\ B_{lat}(s)=\frac{p(s)}{\vartheta (s)}=\frac{L_{\delta_{lat}}}{s+L_{p}},B_{lon}(s)=\frac{q(s)}{a(s)}=\frac{M_{\delta_{lon}}}{s+M_{q}} \end{array}$$

where $\tau_{l}$ is the linkage time delay, $\tau_{f}$ is the rotor-flap time constant, $L_{\delta_{lat}}$ and $M_{\delta_{lon}}$ are the control effectiveness of the rotor’s lateral and longitudinal control, and $L_{p}$ and $M_{q}$ are the damping derivatives. Then, the rotor-body transfer function for identification is derived:

(3) $$\frac{p(s)}{\delta_{lat}(s)}=\frac{L_{\delta_{lat}}e^{-\tau_{l}s}}{\left(s+L_{p}\right)},\frac{q(s)}{\delta_{lon}(s)}=\frac{M_{\delta_{lon}}e^{-\tau_{l}s}}{\left(\tau_{f}s+1)(s+M_{q}\right)}$$

**Figure 7:**
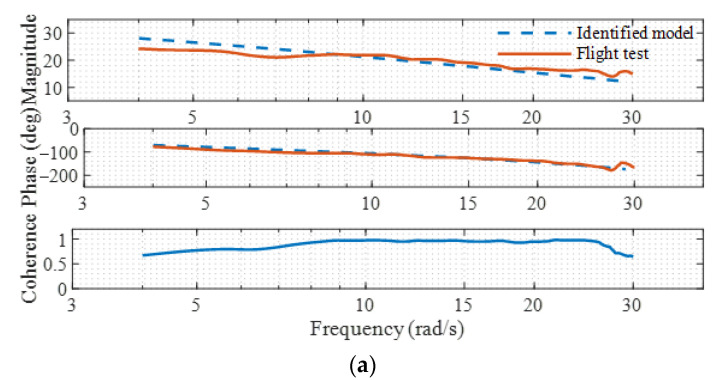

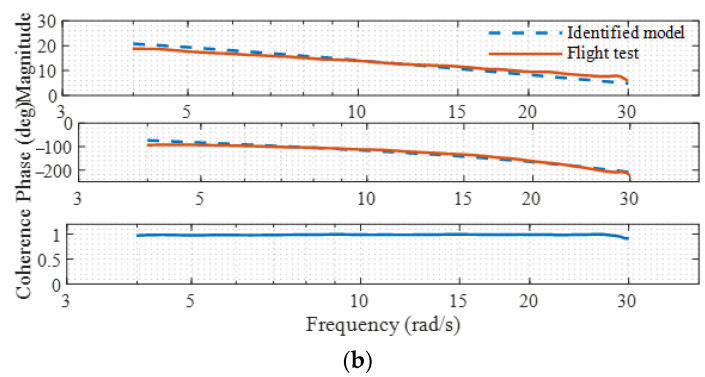
Frequency-domain comparisons. (**a**) Roll channel; (**b**) Pitch channel.

**Figure 8:**
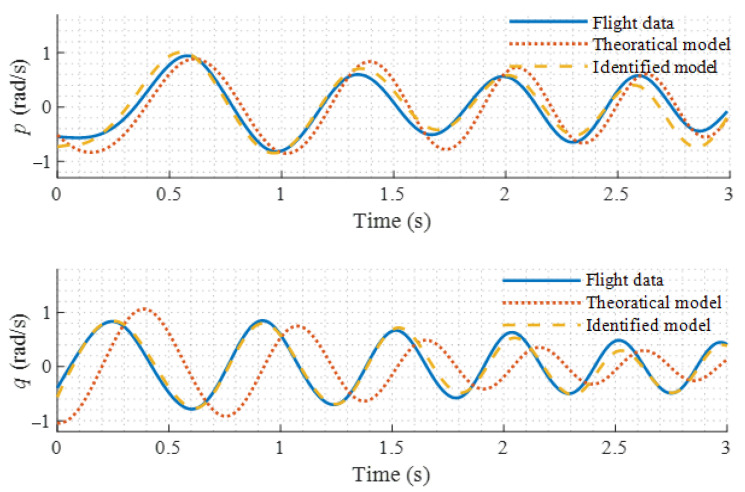
Time-domain comparison.

Frequency-domain system identifications are conducted based on Equation (3) using the recorded control input $\delta_{lon}(t)$, $\delta_{lat}(t)$ and state output q(t), p(t). Frequency-domain identification results are shown in Figure 7. Figure 8 shows the comparison of the time-domain responses of the theoretical model, identified model, and flight data. It can be seen that the coherence of the identification results is close to 1. The frequency-domain responses of the identified model match the flight data with associated cost function values below 100 [31], which indicates that the simplified models have satisfactory fitness. From Figure 8, it can be seen that the time-domain response of the model after identification becomes closer to the flight data. The identified model parameters are listed in Table 2.

## 3. Unified Accurate Attitude Control Using Modified LADRC

**Figure 9:**
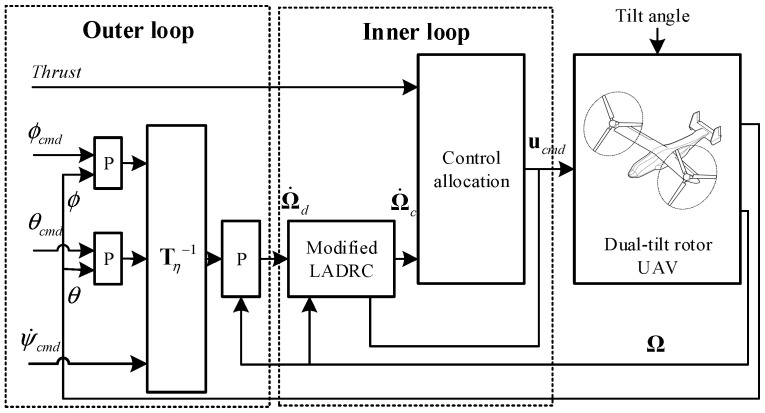
Block diagram of the attitude controller.

The structure of the whole attitude controller is shown in Figure 9. The controller can be divided into two loops. The outer loop generates the desired angular acceleration vector ${\dot{\Omega }}_{d}$ based on the attitude and angular rate errors using cascaded proportional controllers. The inner loop controller consists of a modified LADRC controller and a control allocator. The modified LADRC compensates for the relatively slow rotor flapping dynamics in (2) to avoid oscillation. The LADRC controller forces the plant to track the desired angular acceleration despite disturbances and model uncertainties. Then, according to the angular acceleration and thrust command, the control allocator calculates the control command vector $u_{cmd}$ using the daisy-chain-based control allocation algorithm. 

### 3.1. Actuator Dynamics Compensated LADRC

The rotor flapping dynamics are crucial factors that must be considered when designing the LADRC attitude controller for the dual-tiltrotor UAV that has two helicopter rotors. However, the original LADRC controller design process assumes that the actuator dynamics are fast enough and are ignored [22,32]. The helicopter rotor can be treated as a unique actuator that generates control forces and moments by tilting its tip-path-plane according to its collective and cyclic pitch input. However, the response of helicopter rotors to the collective and cyclic pitch control is relatively slow owing to the inertia and aerodynamics of the blades. If the slow rotor flapping dynamics are ignored, the important assumption for the original LADRC will no longer be valid and the original LADRC will oscillate [24,33,34,35,36]. Thus, the compensation for the rotor flapping dynamics and other actuators is indispensable in the LADRC attitude controller for the dual-tiltrotor UAV.

The aircraft’s aerodynamic surfaces are driven by servos and can be modeled by the first-order transfer function:

(4) $$\frac{\delta_{a}(s)}{\delta_{a}{}_{cmd}(s)}=\frac{\delta_{e}(s)}{\delta_{e}{}_{cmd}(s)}=\frac{\delta_{r}(s)}{\delta_{r}{}_{cmd}(s)}=\frac{1}{\tau_{a}s+1}=A(s)$$

where $\delta_{a}$, $\delta_{e}$, and $\delta_{r}$ are the deflections of the aileron, elevator, and rudder, respectively; $\delta_{a}{}_{cmd}$, $\delta_{e}{}_{cmd}$, and $\delta_{r}{}_{cmd}$ are the commands, respectively; and $\tau_{a}=0.02\;s$ is the servo’s time constant that is given by the servo’s manual. 

As for the rotors, their response to the collective pitch command is modeled by the identified linkage transfer function in (2):

(5) $$\frac{\vartheta_{L}(s)}{\vartheta_{Lcmd}(s)}=\frac{\vartheta_{R}(s)}{\vartheta_{Rcmd}(s)}=e^{-\tau_{l}s}=L(s)$$

where $\tau_{l}$ is the linkage time delay and $\vartheta_{Lcmd}$ and $\vartheta_{Rcmd}$ are the collective pitch commands. The rotor dynamics model consists of the identified linkage and rotor flapping models in (2):

(6) $$\frac{a_{L}(s)}{B_{1Lcmd}(s)}=\frac{a_{R}(s)}{B_{1Rcmd}(s)}=L(s)R(s)=\frac{e^{-\tau_{l}s}}{\tau_{f}s+1}$$

where $B_{1Lcmd}$ and $B_{1Rcmd}$ are the cyclic pitch commands and $a_{L}$ and $a_{R}$ are the longitudinal flapping angles. 

The values of the parameters in (5) and (6) are listed in Table 2. The above rotor and servo models form an actuator model matrix between the control effectors’ deflections and their commands:

(7) $$u(s)=\mathrm{Diag}[L(s),L(s),L(s)R(s),L(s)R(s),A(s),A(s),A(s)]u_{cmd}(s)$$

where $u={\left[\begin{array}{ccccccc} \vartheta_{L}, & \vartheta_{R}, & a_{L}, & a_{R}, & \delta_{a}, & \delta_{e}, & \delta_{r} \end{array}\right]}^{T}$ is the vector of control effectors’ deflections and $u_{cmd}={\left[\begin{array}{ccccccc} \vartheta_{Lcmd}, & \vartheta_{Rcmd}, & B_{1}{}_{Lcmd}, & B_{1}{}_{Rcmd}, & \delta_{acmd}, & \delta_{ecmd}, & \delta_{rcmd} \end{array}\right]}^{T}$ is the controller’s command vector.

The rotational dynamics of the aircraft are derived, whose input is the control effectors’ real deflections:

(8) $$\begin{array}{l} \dot{\eta }=T_{\eta }\Omega \\ \dot{\Omega }=J^{-1}(M_{ext}-\Omega \times J\Omega )+Bu \end{array}$$

where $\eta ={\left[\begin{array}{ccc} \phi , & \theta , & \psi \end{array}\right]}^{T}$ is the Euler angle vector, $\Omega ={\left[\begin{array}{ccc} p, & q, & r \end{array}\right]}^{T}$ is the angular velocity of the aircraft, J is the vehicle moment of inertia tensor, and $M_{ext}$ is the external disturbance moment. B is the control effectiveness matrix relative to the input vector u. $T_{\eta }$ is the transformation matrix between the attitude rate and body angular velocity:

(9) $$T_{\eta }=\left[\begin{array}{ccc} 1 & \sin\phi \tan\theta & \cos\phi \tan\theta \\ 0 & \cos\phi & -\sin\phi \\ 0 & \sin\phi /\cos\theta & \cos\phi /\cos\theta \end{array}\right]$$

Let $B_{0}$ be the nominal value of B and define the total disturbance as

(10) $$f=J^{-1}(M_{ext}-\Omega \times J\Omega )+(B-B_{0})u,$$

Then, the angular acceleration in (8) can be rewritten as

(11) $$\dot{\Omega }=f+B_{0}u$$

To estimate the total disturbance f, a linear extended state observer (LESO) is designed [22,23,32]:

(12) $$\begin{array}{l} \dot{\hat{\Omega }}=\beta_{1}(\Omega -\hat{\Omega })+\hat{f}+B_{0}u\\ \dot{\hat{f}}=\beta_{2}(\Omega -\hat{\Omega }) \end{array}$$

where $\hat{\Omega }$ is the estimation of Ω, $\hat{f}$ is the extended state that represents the estimation of f, and $\beta_{1}$ and $\beta_{2}$ are the tuning parameters of the LESO that determine the bandwidth of the LESO denoted as $\omega_{0}$. They have the following relationships [27]:

(13) $$\beta_{1}=1.41\omega_{0},\beta_{2}=\omega_{0}{}^{2}$$

Different from the original LADRC, the modified LADRC uses the actuator deflections u, other than the command $u_{cmd}$, as the input of the LESO. u is calculated from (7). The LADRC control law is derived:

(14) $$\begin{array}{l} {\dot{\Omega }}_{c}={\dot{\Omega }}_{d}-\hat{f}\\ u_{cmd}=B_{0}{}^{+}{\dot{\Omega }}_{c} \end{array}$$

where ${\dot{\Omega }}_{d}={\left[\begin{array}{ccc} {\dot{p}}_{d}, & {\dot{q}}_{d}, & {\dot{r}}_{d} \end{array}\right]}^{T}$ is the desired angular acceleration vector from the outer loop controller, ${\dot{\Omega }}_{c}={\left[\begin{array}{ccc} {\dot{p}}_{c}, & {\dot{q}}_{c}, & {\dot{r}}_{c} \end{array}\right]}^{T}$ is the angular acceleration vector that the aircraft should generate, and $B_{0}{}^{+}$ represents the inverse of $B_{0}$ calculated by the control allocation algorithm introduced later.

**Figure 10:**
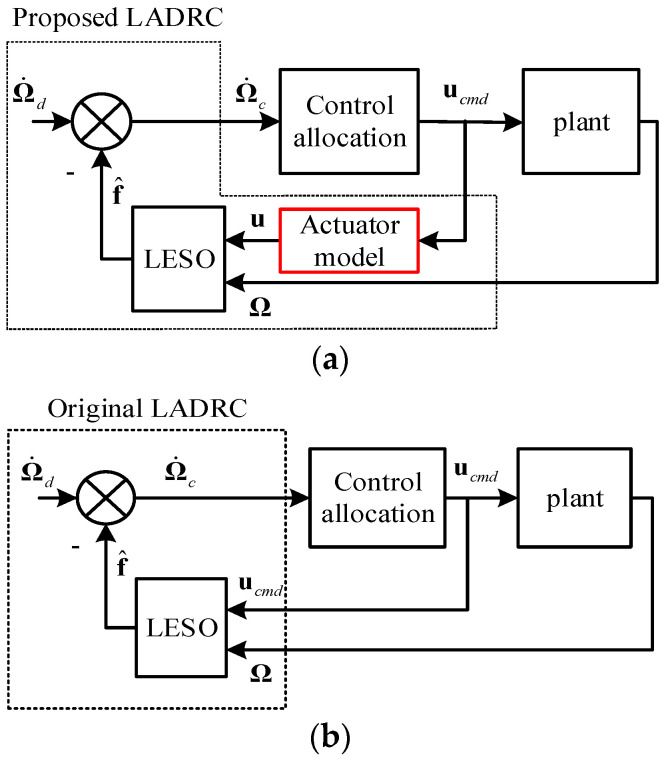
Proposed and original LADRC controller. (**a**) Actuator dynamics compensated LADRC attitude controller; (**b**) Original LADRC attitude controller.

The block diagrams of the proposed and original LADRC controller along with the plant are shown in Figure 10.

It is shown in Figure 10 that the main difference between the proposed and original LADRC controller is the added actuator models described in (4)–(7). Instead of directly feeding $u_{cmd}$ to the LESO, servo and rotor flapping dynamics models are integrated into the controller to synchronize u and Ω, which avoids the oscillations of the closed-loop system.

### 3.2. Daisy-Chain-Based Control Allocation

The studied dual-tiltrotor UAV has redundant controls for the longitudinal and latitudinal control channels. The control allocation for the dual-tiltrotor UAV is more than a mathematical problem of solving an under-determined linear equation with some constraints. Some practical issues should be considered when allocating the redundant controls of the dual-tiltrotor UAV. First, the aerodynamic effectors should be washed out when at low airspeed, because they are easily saturated owing to their low control power, which causes undesirable difficulties in the linear allocation of all control effectors [37]. Second, when at high airspeed, the priority of aerodynamic effectors should be higher than the rotor effectors. Because, in that case, the aerodynamic effectors are faster and more efficient, and the rotor’s cyclic pitch control is limited by the structural strength of the aircraft [29]. Third, the rotor controls for the roll, pitch, and yaw channels are highly coupled when the tilt angle varies. The rotor’s control effectiveness is not only a function of the flight states, but also a function of the nacelle’s tilt angle. This means that it is a strongly time-varying matrix with nontrivial cross-coupling terms among roll, pitch, and yaw channels. Consequently, unlike the aerodynamic effectors, the saturation of one rotor effector will severely affect multiple control channels.

To counteract this problem, a daisy-chain-based [37] control allocator is proposed. In order to decouple the controls for different channels, virtual control effectors are defined and these effectors are divided into two groups: the aerodynamic effectors and rotor effectors, as shown in Table 3 and Table 4. According to the daisy-chain algorithm, aerodynamic effectors are first utilized to generate the desired angular accelerations. The rotor controls are not engaged until the aerodynamic controls are saturated and the commands are not fully satisfied. A washout function is designed to wash out the aerodynamic controls at low airspeed.

The virtual control input vector and real control input vector are partitioned according to the groups:

(15) $$u_{v}={\left[\begin{array}{cc} u_{v}{}_{r} & u_{a} \end{array}\right]}^{T},u={\left[\begin{array}{cc} u_{r} & u_{a} \end{array}\right]}^{T}$$

where $u_{v}={\left[\begin{array}{ccccccc} \delta_{col}, & \delta_{lat}, & \delta_{lon}, & \delta_{dir}, & \delta_{a}, & \delta_{e}, & \delta_{r} \end{array}\right]}^{T}$ is the vector of virtual control effectors, $u_{v}{}_{r}={\left[\begin{array}{cccc} \delta_{col}, & \delta_{lat}, & \delta_{lon}, & \delta_{dir} \end{array}\right]}^{T}$ is the vector of virtual rotor effectors, $u_{r}={\left[\begin{array}{cccc} \vartheta_{L}, & \vartheta_{R}, & B_{1L}, & B_{1R} \end{array}\right]}^{T}$ is the vector of real rotor effectors, and $u_{a}={\left[\begin{array}{ccc} \delta_{a}, & \delta_{e}, & \delta_{r} \end{array}\right]}^{T}$ is the vector of aerodynamic effectors. According to the definitions in Table 4, the relationship between $u_{v}$ and u is

(16) $$u_{v}=\left[\begin{array}{cc} D & 0_{3\times 3}\\ 0_{3\times 3} & I_{3\times 3} \end{array}\right]u,D=\left[\begin{array}{cccc} 0.5 & 0.5 & 0 & 0\\ 0.5 & -0.5 & 0 & 0\\ 0 & 0 & 0.5 & 0.5\\ 0 & 0 & -0.5 & 0.5 \end{array}\right]$$

Therefore, the daisy-chain control allocation problem is to solve the following equation:

(17) $$\begin{array}{ccc} \; & B_{vr}u_{v}{}_{r}+{\left[\begin{array}{cc} 0, & B_{a}u_{a} \end{array}\right]}^{T}={\left[\begin{array}{cc} T_{c}, & {\dot{\Omega }}_{c} \end{array}\right]}^{T},B_{vr}\in R^{4\times 4},B_{a}\in R^{3\times 3} & u_{vr}{}_{\min}\leq u_{vr}\leq u_{vr}{}_{\max},u_{a}{}_{\min}\leq u_{a}\leq u_{a}{}_{\max} \end{array}$$

where $T_{c}$ is the rotor’s thrust command, ${\dot{\Omega }}_{c}$ is the angular acceleration command, $B_{vr}$ is the square effectiveness matrix for virtual rotor effectors, and $B_{a}$ is the square effectiveness matrix for aerodynamic effectors. 

According to the daisy-chain algorithm, $u_{a}$ and $u_{v}{}_{r}$ are calculated sequentially in the two allocation steps. Firstly, the deflections of the aerodynamic effectors are derived:

(18) $$\begin{array}{l} {\dot{\Omega }}_{ca}=K_{w}(v_{a}){\dot{\Omega }}_{c}\\ u_{a}=sat(B_{a}^{-1}{\dot{\Omega }}_{ca}) \end{array},\;K_{w}(v_{a})=\{\begin{array}{cc} 0 & 0\leq v_{a}<v_{1}\\ \frac{v_{a}-v_{1}}{v_{2}-v_{1}} & v_{1}\leq v_{a}<v_{2}\\ 1 & v_{a}\geq v_{2} \end{array}$$

where ${\dot{\Omega }}_{ca}$ is the angular acceleration command for the aerodynamic effectors, $v_{a}$ is the airspeed, $K_{w}(v_{a})$ is the washout function, and $v_{1}$ and $v_{2}$ are the airspeeds at which the washout function begins and ends. sat(·) represents a saturation function that makes sure all effectors are within their deflection limits by cutting the results that are out of bounds. Take an input vector with *n* elements, for example, it works as

(19) $$\begin{array}{c} sat(u)={\left[\begin{array}{cccc} s(u_{1}), & s(u_{2}), & ... & s(u_{n}) \end{array}\right]}^{T}\\ s(u_{i})=\{\begin{array}{cc} u_{i\min} & u_{i}\leq u_{i\min}\\ u_{i} & u_{i\min}\leq u_{i}\leq u_{i\max}\\ u_{i\max} & u_{i}\geq u_{i\max} \end{array},(i=1,2,\ldots n) \end{array}$$

where $u_{i\min}$ and $u_{i\max}$ are the element’s lower and upper limits. As the aerodynamic effectors are decoupled among the control channels, this simple anti-windup function is reasonable and practical.

Next, the rotor’s angular acceleration command and the thrust command are satisfied by the virtual rotor effectors $u_{v}{}_{r}$. The second control allocation step is to solve:

(20) $$\begin{array}{c} {\dot{\Omega }}_{cr}={\dot{\Omega }}_{c}-B_{a}u_{a},B_{vr}u_{v}{}_{r}={\left[\begin{array}{cc} T_{c} & {\dot{\Omega }}_{cr} \end{array}\right]}^{T}\\ u_{vr}{}_{\min}\leq u_{vr}\leq u_{vr}{}_{\max} \end{array}$$

where ${\dot{\Omega }}_{cr}$ is the angular acceleration command for the virtual rotor effectors.

**Figure 11:**
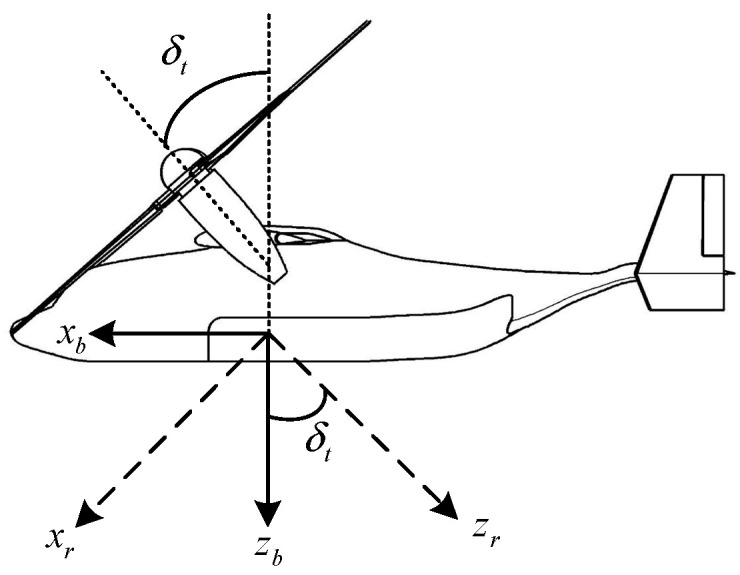
Rotor mast axes.

The rotor effectiveness matrix $B_{vr}$ is resolved to the rotor mast axes [38] to simplify the calculation. The rotor mast axes are illustrated in Figure 11, where the subscript b represents the body axes and the subscript r represents the rotor mast axes. Its origin coincides with the body axes. It is obtained by rotating the body axes along its *y*-axis in its negative direction by $\delta_{t}$. 

The transformation matrix from the body axes to the rotor mast axes is as follows:

(21) $$T_{rb}=\left[\begin{array}{ccc} \cos(\delta_{t}) & 0 & \sin(\delta_{t})\\ 0 & 1 & 0\\ -\sin(\delta_{t}) & 0 & \cos(\delta_{t}) \end{array}\right]$$

Resolving ${\dot{\Omega }}_{cr}$ to the rotor mast axes yields

(22) $${{\dot{\Omega }}^{\prime }}_{cr}=T_{rb}{\dot{\Omega }}_{cr}$$

where ${{\dot{\Omega }}^{\prime }}_{cr}={\left[\begin{array}{ccc} p^{\prime } & q^{\prime } & r^{\prime } \end{array}\right]}^{T}$ is the angular acceleration command resolved to the rotor mast axes. The nominal control effectiveness expressed in the rotor mast axes is denoted as ${B^{\prime }}_{vr}$, and it is a diagonal matrix:

(23) $${B^{\prime }}_{vr}=\mathrm{Diag}(\begin{array}{cccc} \frac{\partial T}{\partial \delta_{col}}, & \frac{\partial {\dot{p}}^{\prime }}{\partial \delta_{lat}}, & \frac{\partial {\dot{q}}^{\prime }}{\partial \delta_{lon}}, & \frac{\partial {\dot{r}}^{\prime }}{\partial \delta_{dir}} \end{array})$$

It is shown from Equation (23) that the control of each virtual rotor effector is decoupled in the rotor mast axes. The relatively trivial cross-coupling terms in the real effectiveness matrix are treated as the disturbances in (10) and compensated by the LADRC controller. Furthermore, (20) becomes the following:

(24) $$\begin{array}{c} {\dot{\Omega }}_{cr}={\dot{\Omega }}_{c}-B_{a}u_{a},{{\dot{\Omega }}^{\prime }}_{cr}=T_{rb}{\dot{\Omega }}_{cr},{B^{\prime }}_{vr}u_{v}{}_{r}={\left[\begin{array}{cc} T_{c} & {{\dot{\Omega }}^{\prime }}_{cr} \end{array}\right]}^{T}\\ u_{vr}{}_{\min}\leq u_{vr}\leq u_{vr}{}_{\max} \end{array}$$

Then, the deflections of virtual rotor effectors are derived based on (24).

(25) $$u_{v}{}_{r}=sat(B_{vr}^{-1}{\left[\begin{array}{cc} T_{c} & {{\dot{\Omega }}^{\prime }}_{cr} \end{array}\right]}^{T})$$

The final deflection commands for the real controls are derived using (16):

(26) $$u_{cmd}={\left[\begin{array}{cc} D & 0_{3\times 3}\\ 0_{3\times 3} & I_{3\times 3} \end{array}\right]}^{-1}\left[\begin{array}{c} u_{vr}\\ u_{a} \end{array}\right]$$

**Figure 12:**
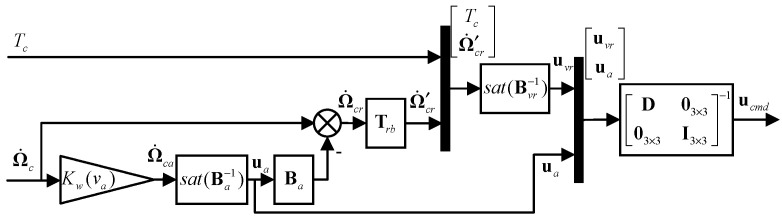
Block diagrams for the daisy-chain-based control allocation.

The overall daisy-chain-based control allocation process is shown in Figure 12. 

## 4. Simulation Results

The purpose of the simulation is to evaluate the effectiveness of the modification in LADRC and verify the robustness and performance of the proposed controller in different flight modes. The aircraft dynamics model is based on the dual-tiltrotor UAV used in the flight tests. The model is built via theoretical modeling and system identification, as described in Section 3. As shown in Figure 9, the main controller parameters of the LADRC include the outer loop gains and the inner loop control effectiveness matrix ***B*** in (8). The inner loop control effectiveness matrix is obtained by the system identification described in Section 2.3. Once the model related parameter ***B*** is determined, the outer loop gains are designed via frequency-domain tools according to the handling quality requirements, for example, the cross-over frequency and damping ratio of the control system.

### 4.1. Effectiveness of the Modified LADRC Controller

**Figure 13:**
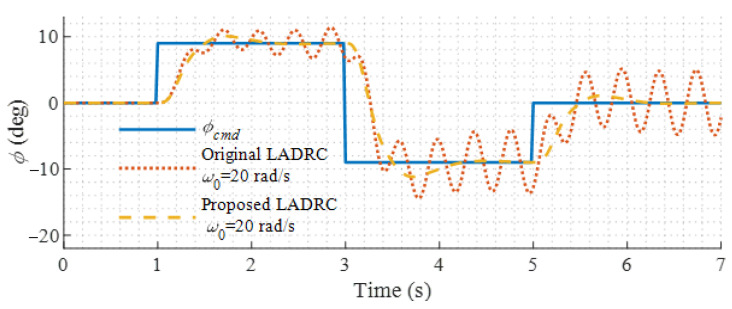
Comparison of original and proposed LADRC.

The command tracking performance of the modified LADRC controller and the original LADRC controller is compared. In the helicopter mode, standard doublet roll and pitch commands are given to the modified and original LADRC controllers. Figure 13 shows their responses.

As shown in Figure 13, the roll and pitch responses of the original LADRC controller have severe oscillation. However, the modified LADRC controller has the desired responses. It can be seen that the rotor flapping dynamics and the actuator dynamics of the dual-tiltrotor UAV have non-trivial effects on the stability of the closed-loop control system and cannot be neglected in the design of the LADRC controller. With the help of the added actuator model, the actuator feedback signals are synchronized with the state feedback signals before they are fed to the LESO, and the oscillation disappears. Thus, the effectiveness of the modified LADRC is verified.

**Figure 14:**
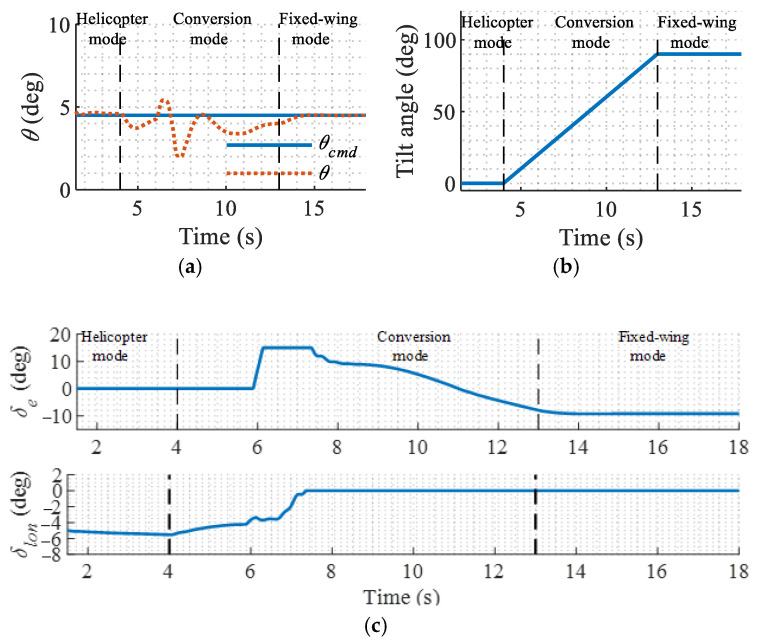
Conversion flight simulation results. (**a**) Pitch angle; (**b**) Tilt angle; (**c**) Elevator and cyclic pitch response.

Conversion flight tests from the helicopter mode to the fixed-wing mode are conducted in the simulation using the proposed controller. The results are shown in Figure 14.

The controller ensures stability in different flight modes. The control allocator washes out the deflections of the aerodynamic control surfaces in the low-speed flight period and washes out the rotor’s cyclic pitch control in the high-speed flight period, which guarantees the control efficiency in different flight modes.

**Figure 15:**
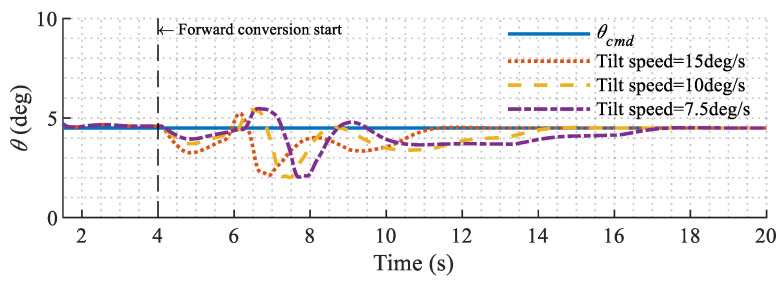
Simulation results with different tilt speeds.

To better verify that the aircraft is stabilized when the tilt angle changes at different speeds, more conversion flight simulations are conducted with different tilt speeds. The pitch responses are shown in Figure 15. Overall, the pitch angle tracking error is within ±3° and the tilt speed has minor effects on the performance of the controller. 

### 4.2. Robustness against Model Uncertainties and Disturbances

**Figure 16:**
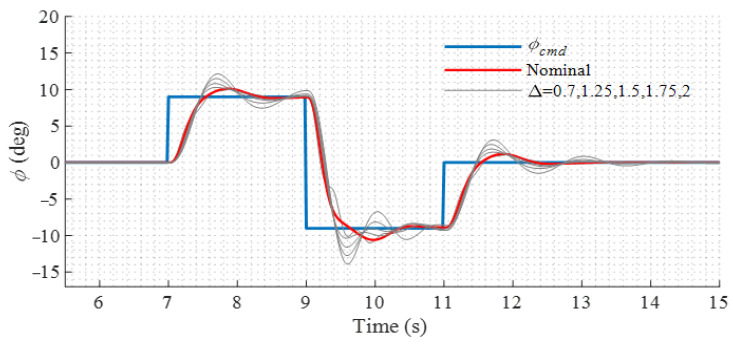
Roll responses in conversion mode under model uncertainties.

Though the LADRC controller relies on fewer model parameters than conventional model-based controllers, it is sensitive to the control effectiveness introduced in (8) and (13). Simulations are conducted to demonstrate the robustness of the proposed controller against the uncertainties of the control effectiveness. The nominal control effectiveness matrix is denoted as $B_{0}$. In the simulations, perturbations are added to the control effectiveness of the model, which is presented as

(27) $$B=\Delta B_{0}\left(\Delta =0.7,1.25,1.5,1.75,2\right)$$

where B is the control effectiveness of the model and Δ is the perturbation coefficient. The roll responses to the doublet command in the conversion mode where the tilt angle is 45° are shown in Figure 16. It can be seen that the controller remains stable despite the fact that the model parameters vary over a relatively wide range.

**Figure 17:**
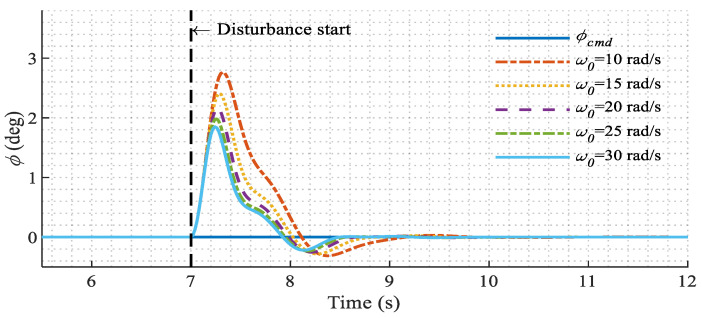
Roll responses in conversion mode under external disturbances.

The robustness against external disturbances is also examined in the simulation. In the conversion mode where the tilt angle is 45°, a constant disturbance roll moment is applied to the model. The responses with different LESO bandwidth $\omega_{0}$ are compared in Figure 17. It is shown that, as $\omega_{0}$ increases, the settling time becomes shorter and the peak value becomes smaller. This result reveals one of the advantages of the proposed modified LADRC over the original one. That is, with the help of the compensated rotor dynamics, the bandwidth of the LESO is extended and higher robustness against disturbances is achieved.

## 5. Flight Test Results

**Figure 18:**
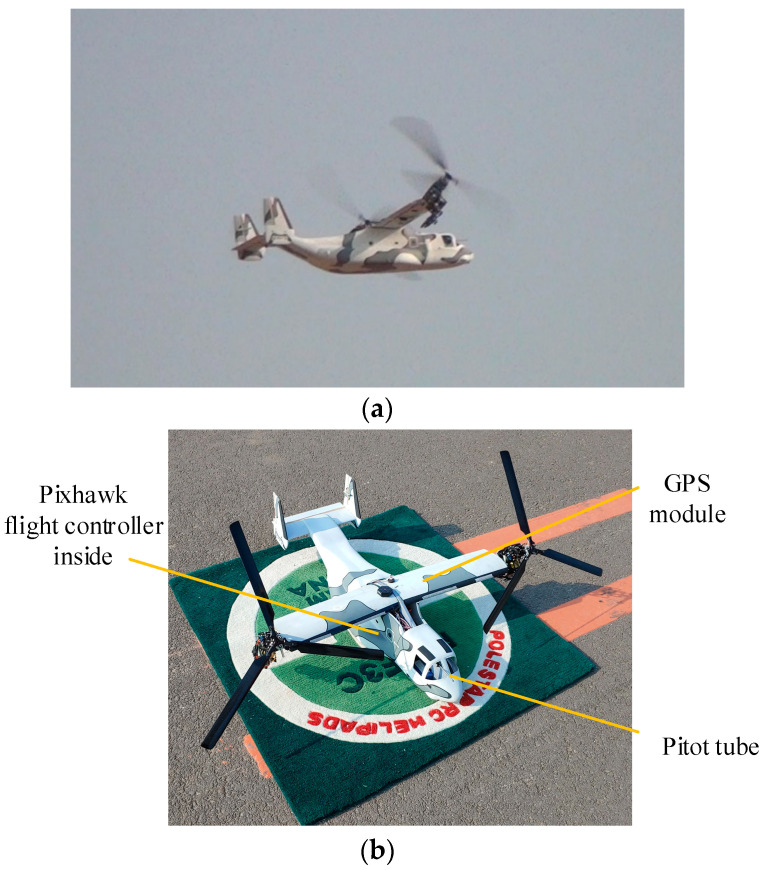
Dual-tiltrotor UAV in flight tests. (**a**) UAV in flight tests; (**b**) Main flight control devices.

The dual-tiltrotor UAV used in the flight tests is a modified RC model aircraft (a.k.a. Rotormast Scale RC VTOL), and it is shown in Figure 18. The original model aircraft only has collective and cyclic pitch controls and lacks aerodynamic control surfaces. Thus, the frame of the aircraft is modified and ailerons, elevator, rudders, and servos are added on the trailing edges of its wings and tails. The original flight controller is replaced by a Pixhawk flight controller and supplementary avionics like GPS and Pitot tube, as shown in Figure 18. The control laws are implemented into the flight controller, which runs PX4 open-source software. The aircraft parameters are listed in Table 5. The inertia tensor of the studied UAV is obtained via the trifilar pendulum approach [39].

### 5.1. Comparison of Modified LADRC with PID Controller

First, the performance of the proposed controller is compared with a PID controller in the helicopter mode. The PID controller is designed and tuned theoretically via frequency-domain and time-domain simulations. Then, it is fine-tuned using the empirical method in the flight tests. Designing a safe PID controller for the entire flight envelope is difficult owing to the large model uncertainty of the studied dual-tilt rotor UAV. Therefore, the PID controller is only compared in the helicopter mode.

**Figure 19:**
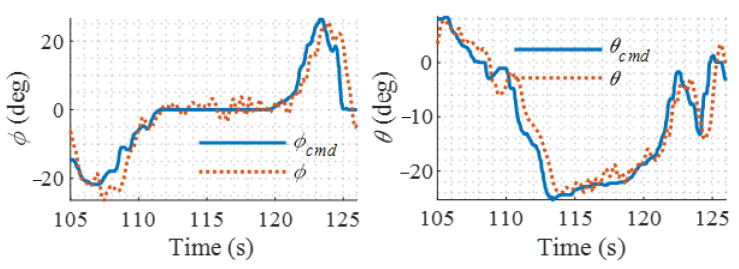
Hover flight test results for modified LADRC.

**Figure 20:**
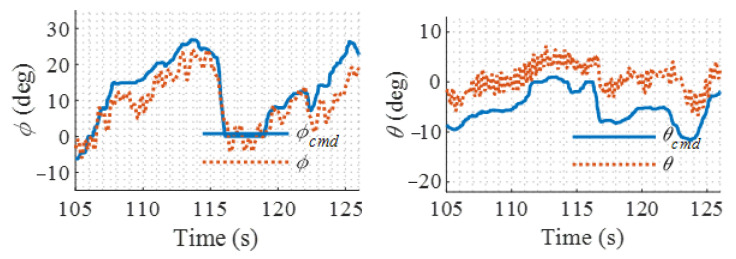
Hover flight test results for PID.

The flight results of the proposed LADRC controller and PID controller are shown in Figure 19 and Figure 20, respectively. It is shown that the proposed controller has higher attitude command tracking accuracy than the PID controller. Large static error appears in the pitch channel when using PID controller owing to the saturation of the integrators. The PID controller has a bigger attitude fluctuation during the hover flight than the proposed controller. This is because the modified LADRC controller can estimate the external disturbances by the LESO and then compensate for their effects. Therefore, the proposed controller has a faster settling speed against disturbances than the PID controller. On the other hand, the PID controller uses the integration of the errors in attitude angles or angular rates to compensate for the disturbance. Further, its integrator gain is limited by the stability requirement of the system, which means a trade-off must be made between the stability and disturbance rejection capability during the design of the PID controller. The small integration gain guarantees stability, but a big tracking error is its cost.

### 5.2. Performance of Modified LADRC in Different Flight Modes

**Figure 21:**
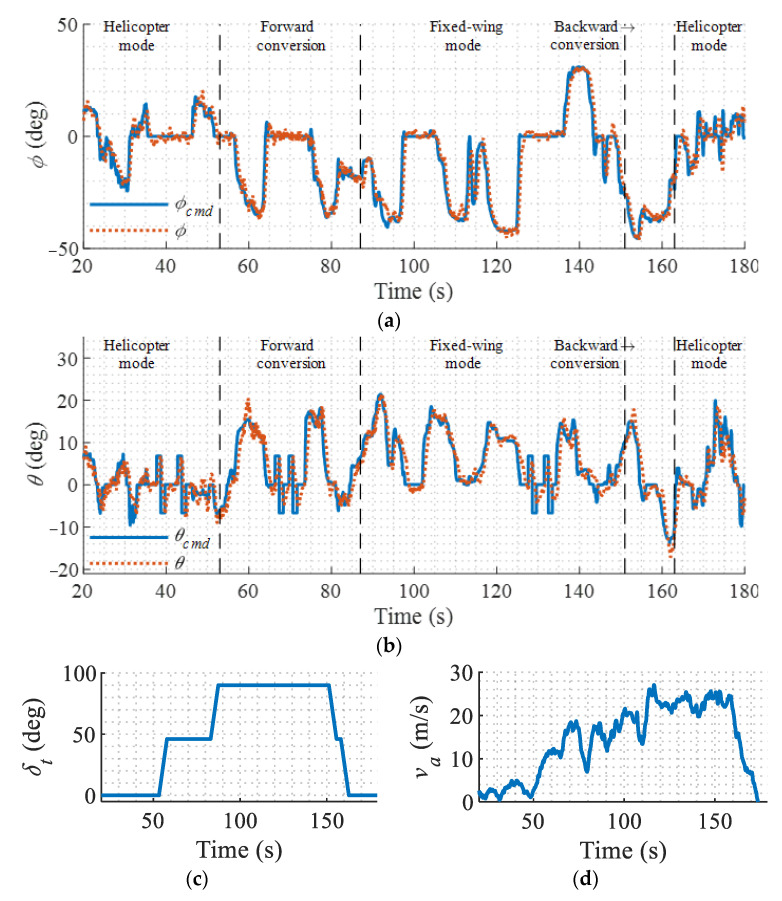
Flight results in forward and backward conversion flight. (**a**) Roll angle response; (**b**) Pitch angle response; (**c**) Tilt angle; (**d**) Airspeed.

To examine the performance of the proposed controller in the entire flight envelope, a typical conversion flight is conducted. The aircraft takes off in the helicopter mode and converts to the fixed-wing mode, then converts back to the helicopter mode. The flight results of the entire process are shown in Figure 21.

**Figure 22:**
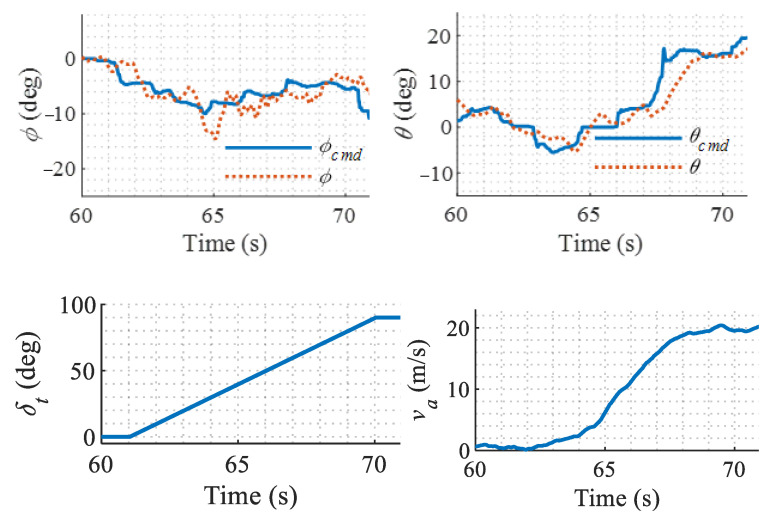
Responses in continuous forward conversion flight.

It can be seen from Figure 21 that, although the airspeed, the configuration of the aircraft, and available controls have dramatic changes in different flight modes, the controller keeps the aircraft stable with satisfactory attitude tracking performance. Another set of flight results are shown in Figure 22, which presents more clearly the responses during a continuous forward conversion flight from the helicopter mode to the fixed-wing mode. It is shown that the attitude tracks the command with a mean error of less than ±4° despite the chittering caused by the sensor measurement noise and mechanical jitter. With the help of the modification in LADRC, the relatively slow rotor flapping dynamics and servo dynamics are compensated; therefore, the severe oscillation of the original LADRC is avoided and the stability is guaranteed.

**Figure 23:**
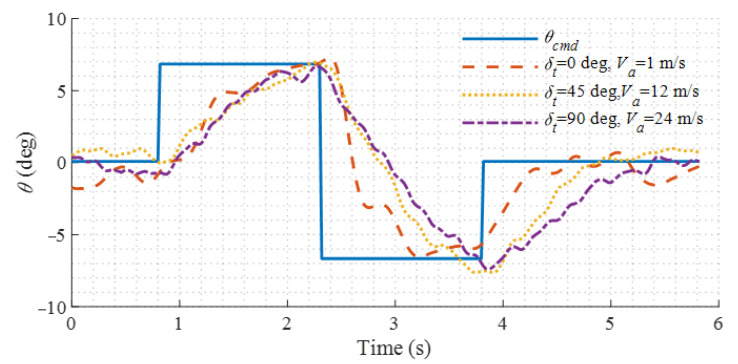
Pitch responses to the standard command in different flight modes.

To further evaluate the variation of controller performance in different flight modes, a standard doublet pitch angle command is given when the aircraft is in helicopter mode, conversion mode, and fixed-wing mode, respectively. The pitch angle responses in different flight modes are extracted and compared in Figure 23. It can be seen that the control rising time in different flight modes is all about 0.5 s, despite the variations in the airspeed, tilt angle, and control effectors in use. Slight differences occur when the pitch angle was restored to the neutral position. This may be because the damping effect of the wing and fuselage is different at different airspeeds.

## 6. Conclusions

In this paper, a unified attitude controller based on the modified LADRC is proposed for a dual-tiltrotor UAV to counteract its nonlinearity, time-varying dynamics, and model mismatch during flight mode transitions. The proposed control algorithm has higher robustness against model mismatch compared with the model-based control algorithms. It has less dependency on the accurate dynamics model of the dual-tiltrotor UAV, which can hardly be built. Future work will focus on combing the adaptive control technique with the proposed controller to identify the model related control parameters online. In this way, the robustness of the controller can be further improved. 

In contrast to the original LADRC, an actuator model is integrated into the modified LADRC to compensate for the non-negligible slow rotor flapping dynamics and servo dynamics. This modification eliminates the oscillation of the original LADRC when applied on the plant with slow-response actuators, such as propeller and rotors of the helicopter. As a result, the stability and performance of the LADRC controller are improved compared with the original LADRC. 

The simulation and flight test results show that the modified LADRC controller has high stability and control accuracy in the entire flight envelope despite the change in the tilt angle and airspeed. The oscillation of the original LADRC controller is also avoided. The attitude control error is less than ±4° during the conversion flight. Moreover, the flight results show that there are only minor differences in control performance between different flight modes. The control rising time of the step response in different flight modes is about 0.5 s in all cases, despite intense variations in the airspeed, tilt angle, and control effectors in use. The flight results show that the controller guarantees high control accuracy and uniform control quality in different flight modes.

## Figures and Tables

**Table 1:** Controls of the aircraft.

	Name	Symbol	Units
Rotor controls	Left collective pitch	$\vartheta_{L}$	Rad
	Right collective pitch	$\vartheta_{R}$	Rad
	Left longitudinal cyclic pitch	$B_{1L}$	Rad
	Right longitudinal cyclic pitch	$B_{1R}$	Rad
Aerodynamic surfaces	Aileron	$\delta_{a}$	Rad
	Elevator	$\delta_{e}$	Rad
	Rudder	$\delta_{r}$	Rad

**Table 2:** Identification results.

Symbol	Value	Units	Insensitivity
$L_{p}$	−2.79	-	5.08%
$M_{q}$	−2.62	-	4.85%
$L_{\delta_{lat}}$	122.00	-	1.38%
$M_{\delta_{lon}}$	52.18	-	1.30%
$\tau_{l}$	0.020	s	1.37%
$\tau_{f}$	0.052	s	2.00%

**Table 3:** Decoupled virtual control effectors.

	Name	Symbol	Definition	Units
Virtual rotor effectors	Symmetric collective pitch	$\delta_{col}$	$0.5(\vartheta_{L}$$+\vartheta_{R}$)	Rad
	Asymmetric collective pitch	$\delta_{lat}$	$0.5(\vartheta_{L}$$-\vartheta_{R}$)	Rad
	Symmetric cyclic pitch	$\delta_{lon}$	$0.5(B_{1L}$$+B_{1R}$)	Rad
	Asymmetric cyclic pitch	$\delta_{dir}$	$-0.5(B_{1L}$$-B_{1R}$)	Rad
Aerodynamic effectors	Aileron	$\delta_{a}$	-	Rad
	Elevator	$\delta_{e}$	-	Rad
	Rudder	$\delta_{r}$	-	Rad

**Table 4:** Control effectors in different modes.

Control Channel	Helicopter Mode	Conversion Mode	Fixed-Wing Mode
Rotor thrust	$\delta_{col}$	$\delta_{col}$	$\delta_{col}$
Roll	$\delta_{lat}$	$\delta_{col}$ $+\delta_{lat}$ $+\delta_{a}$	$\delta_{a}$
Pitch	$\delta_{lon}$	$\delta_{lon}$ $+\delta_{e}$	$\delta_{e}$
Yaw	$\delta_{dir}$	$\delta_{lat}$ $+\delta_{dir}$ $+\delta_{r}$	$\delta_{r}$

**Table 5:** Aircraft parameters.

Parameter	Unit	Value
Mass	kg	3.2
Length	m	0.974
Wingspan	m	1.057
Rotor radius	m	0.32
Inertia tensor (In helicopter mode)	kg·m^2^	$\left[\begin{array}{ccc} 0.825 & 0 & -0.125\\ 0 & 0.638 & 0\\ -0.125 & 0 & 0.896 \end{array}\right]$

## Data Availability

Not applicable.
